# The Effects of Earphone Use and Environmental Lead Exposure on Hearing Loss in the Korean Population: Data Analysis of the Korea National Health and Nutrition Examination Survey (KNHANES), 2010–2013

**DOI:** 10.1371/journal.pone.0168718

**Published:** 2016-12-28

**Authors:** Da-An Huh, Yun-Hee Choi, Kyong Whan Moon

**Affiliations:** 1 Department of Public Health Sciences, Graduate School, Korea University, Seoul, Republic of Korea; 2 Department of Health and Environmental Science, College of Health Science, Korea University, Seoul, Republic of Korea; Chang Gung Memorial Hospital Kaohsiung Branch, TAIWAN

## Abstract

**Background:**

Although previous studies have reported that frequent earphone use and lead exposure are risk factors for hearing loss, most of these studies were limited to small populations or animal experiments. Several studies that presented the joint effect of combined exposure of noise and heavy metal on hearing loss were also mainly conducted on occupational workers exposed to high concentration.

**Objectives:**

We investigated both the individual and joint effects of earphone use and environmental lead exposure on hearing loss in the Korean general population.

**Methods:**

We analyzed data from 7,596 Koreans provided by the Korea National Health and Nutrition Examination Survey (KNHANES) during the period 2010–2013. The pure-tone average (PTA) of hearing thresholds at 2, 3, and 4 kHz frequencies was computed, and hearing loss was defined as a PTA ≥ 25 dB in one or both ears.

**Results:**

A dose-response relationship in hearing loss with earphone use time and blood lead level is observed after adjustment for confounding factors. With a 1-hour increase in earphone use time and 1 μg/dL increase in blood lead concentration, the odds of hearing loss increased by 1.19 and 1.43 times, respectively. For hearing loss, the additive and multiplicative effect of earphone use and blood lead level were not statistically significant.

**Conclusions:**

Earphone use and environmental lead exposure have an individual effect on hearing loss in the general population. However, the estimated joint effect of earphone use and lead exposure was not statistically significant.

## Introduction

Hearing loss is one of the most common chronic disabling conditions [1]. The World Health Organization (WHO) reported that more than 5.3% (360 million people) of the world’s population suffers from hearing loss [2].

Loud noise is a major risk factor for hearing loss, and employees exposed to loud noises in industrial workplaces have been established as a high-risk group [1, 3]. However, with the recent increasing supply of smartphones and MP3 players, the usage of personal sound equipment (earphone) tends to also increase [4, 5], and this increment causes the population to be exposed to non-occupational noise. Long-term usage of earphone could induce hearing loss [4, 6], and a few studies have suggested that hearing loss could occur from earphone overuse, regardless of occupational noise exposure [7, 8].

Ototoxic chemicals cause hearing loss [9–11] and also enhance the extent of hearing loss due to noise [12]. Jones et al. suggested that lead (Pb) is an ototoxic heavy metal and that exposure induces degeneration of inner ear receptor cells and decreases in auditory nerve conduction [13].

Many previous studies have presented evidences that noise and lead exposure could effect on hearing loss. However, most of those studies analyzed small size study population [14–17]. Few epidemiological studies have studied the correlation between hearing loss and frequent earphone use or environmental lead exposure in the general population. And some studies have reported the joint effect of combined exposure to noise and lead exposure on hearing loss [18, 19]. These studies, however, are mainly conducted with industrial workers, therefore the results are difficult to be generalized. Thus, this hearing loss study examined in a sample of the general population is meaningful.

This study investigated the individual and joint effects of earphone use and environmental lead exposure on hearing loss in the Korean population using data from the Korea National Health and Nutrition Examination Survey (KNHANES).

## Materials and Methods

### Study population

The KNHANES is a nationwide survey that represents the general South Korean population and constitutes an ongoing series of cross-sectional surveys. KNHANES provides health-related information such as the health status, lifestyle, and sociodemographic characteristics of participants.

**Fig 1:**
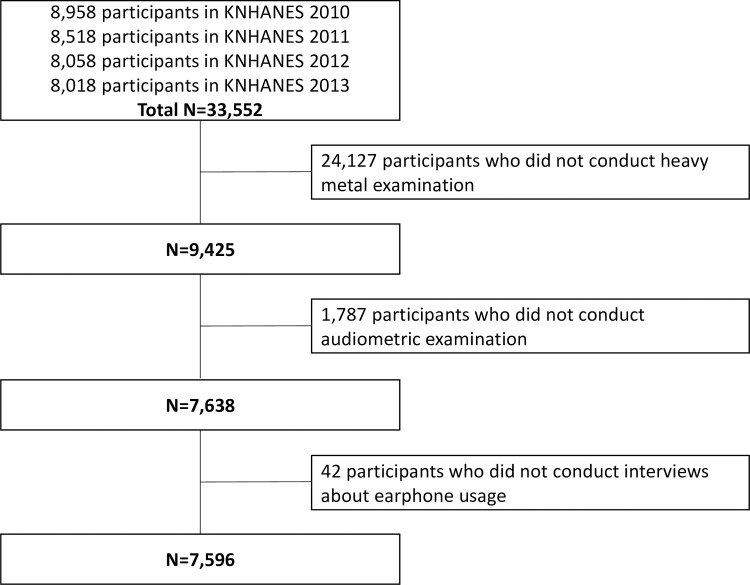
Study population (KNHANES, Korea National Health and Nutrition Examination Survey, 2010–2013).

The KNHANES conduct the audiometric examinations in 2010–2013. We combined 33,552 data from the surveys of 2010–2013 and excluded 25,956 participants who did not have heavy metal or audiometric examinations and interviews regarding earphone use (Fig 1). We selected data from 7,596 participants aged between 10 and 87 for analysis.

The KNHANES survey sample is representative of the general population of South Korea because it uses a stratified multistage probability sampling method based on National Census data. The amount of 7,596 participants represents 36,422,173 Koreans (weighted). S1–S7 Files of supporting information are the raw data of the KNHANES 2010–2013.

### Blood lead levels

Lead (Pb) levels in blood were measured by graphite furnace atomic absorption spectrometry (GFAAS, AAnalyst 600; PerkinElmer, Finland). Analytical equipment was controlled using standard reference material from Whole Blood Metals Control (BIO-RAD, USA) for internal quality assurance, and external quality assurance was satisfied by the German External Quality Assessment (G-EQUAS). The detection limit for Pb was 0.223 μg/dL in the 2010–2013 KNHANES.

### Earphone use

Participant response regarding earphone use was obtained from a self-reported questionnaire survey. Participants were asked if they used earphones daily, and if so, what the average amount of use time was.

### Audiometric measurement

Audiometric examinations were conducted by the pure-tone audiometric testing method. Testing was performed in a sound-isolation booth inside a mobile examination center. Otolaryngologists performed all the audiometric examinations. The pure-tone audiometric testing was conducted by an audiometer (model GSI SA-203; LenaNodin, Sweden) and automated testing was programmed following a modified Hughson–Westlake procedure. Test frequencies were 0.5, 1, 2, 3, 4, and 6 kHz. Participants responded by pushing a button when they heard a tone. In order to choose the frequencies that are used for pure-tone average (PTA), we examined which frequencies are affected by environmental lead exposure. S1 Fig of supporting files presents the hearing thresholds of each frequency by blood lead level quintiles. In 0.5, 1, and 2 kHz, there was no striking difference in hearing thresholds between the groups. In 3 and 4 kHz, however, the hearing thresholds rose as the lead quintile levels increased. In other words, we may underestimate the actual effect of lead on hearing loss when we use common methods of 0.5, 1, 2, and 4 kHz. Therefore, we computed the PTA of hearing thresholds at frequencies 2, 3, and 4 kHz, consistent with the definition of standard threshold shift used by the Occupational Safety and Health Administration (OSHA), which may have better reflection on the hearing abilities at frequencies 3 and 4 kHz. Hearing loss was defined as a PTA ≥ 25 dB in one or both ears according to the definition used by the World Health Organization (WHO).

### Confounding variables

We used demographic and hearing-related variables to control for potentially confounding variables. The variables considered were age, sex, smoking status, monthly income, education levels, body mass index (BMI), occupational noise exposure, loud noise exposure, firearm noise exposure, hypertension, and diabetes mellitus. Smoking status was classified as nonsmoker, past-smoker, or current-smoker, and BMI was calculated as weight (kg)/height (m) squared. Occupational, loud, and firearm noise exposure types were used to classify the exposed and non-exposed groups. Participants were categorized in the exposed to occupational noise group if they reported exposure to noise for over three months in the workplace. The exposed to loud noise group consisted of those who reported exposure to loud noise outside the workplace (e.g., car horn sounds, machine noise, and loud music) for more than 5 hours a week. Participants who reported exposure to firearm noise were classified in the exposed to firearm noise group. Hypertension was diagnosed if the results of a blood pressure test had systolic blood pressure ≥ 140 mmHg or diastolic blood pressure ≥ 90 mmHg at the time of the examination. Diabetes was identified when participants reported a prior physician diagnosis.

### Ethics Statement

The Korean National Health and Nutrition Examination Survey (KNHANES) obtained a written informed consent from each participant prior to conducting the survey, and we used this secondary data for the epidemiology study.

### Statistical analysis

KNHANES was analyzed in consideration of the stratified multistage probability sampling design and weighted sample values used to provide a participant sample representative of the general South Korean population. We calculated an integrated weight value using the combined 2010–2013 KNHANES dataset and applied it to the statistical analysis.

We used a logarithmic transformation of blood lead concentration and PTA to normalize the distribution. The Student t-test and Wald F-test were used to evaluate the difference between geometric mean and arithmetic mean among the groups.

Logistic regression analysis was conducted to estimate an odds ratio (OR) for hearing loss defined as PTA ≥ 25 dB in either ear. Blood lead level and earphone use time data were divided into quintiles, and the OR was estimated by comparing each value in the quintile to the lowest quintile. We developed a sequence of three models to identify influence from potential confounders: a) model A was adjusted for demographic variables such as age, sex, monthly income (<1, 1–2, 2–3, ≥3 million Won), education level (<high school, high school, >high school), smoking status (never-smoked, past-smoker, current-smoker), BMI (continuous), and earphone use status (non-user, user) in blood lead level model and blood lead level (continuous) in earphone use time model; b) model B additionally adjusted for noise-related variables such as occupational noise exposure (non-exposure, exposure), loud noise exposure (non-exposure, exposure), and firearm noise exposure (non-exposure, exposure); and c) model C additionally adjusted for disease variables related to hearing loss such as hypertension (no, yes) and diabetes (no, yes).

Joint effects of earphone use time and blood lead concentration on hearing loss were examined after adjusting for all variables used in model C. We used combinations of categorical variables and classified them into four groups: low use time and low lead (reference); low use time and high lead; high use time and low lead; and high use time and high lead. High and low categories were defined by the median values of each variable. We used two scales of the joint effect, an additive scale [referred to as relative excess risk due to interaction (RERI)] and a multiplicative scale (the ratio of ORs), following the methods recommended by Knol and VanderWeele [20]. We computed the 95% confidence interval (CI) for the RERI following the standard delta method based on a Taylor Series expansion [21].

All statistical analyses were performed using SPSS version 22.0, and the statistical significance level was a two-sided p-value <0.05.

## Results

Table 1 shows the characteristics of the study population. Weighted arithmetic mean (AM) and 95% CI of earphone using time is 1.37 (1.25, 1.49) hours and weighted geometric means (GMs) and 95% CI of blood lead level and PTA are 2.08 (2.05, 2.11) μg/dL and 12.84 (12.40, 13.28) dB, respectively. Earphone using time is not significant in sex and age. Blood lead level is higher in males (p < 0.001) and increases with age (p < 0.001). PTA is also higher in males (p < 0.001) and increases with age (p < 0.001).

**Table 1:** Age-adjusted arithmetic and geometric means and 95% CI of variables by participant characteristics

Variables		Earphone using time (hour)		Blood lead (ug/dL)		Pure-tone average (dB)	
		n = 1036		n = 7596		n = 7596	
		AM (95% CI)^a^	p-value^b^	GM (95% CI)^a^	p-value^b^	GM (95% CI)^a^	p-value^b^
Total		1.37 (1.25, 1.49)		2.08 (2.05, 2.11)		12.84 (12.40, 13.28)	
Sex							
	Male	1.45 (1.27, 1.64)	0.174	2.42 (2.38, 2.46)	<0.001	15.21 (14.71, 15.73)	<0.001
	Female	1.28 (1.11, 1.44)		1.78 (1.75, 1.81)		10.68 (10.28, 11.08)	
Age (years)							
	<20	1.43 (1.18, 1.68)	0.130	1.31 (1.27, 1.35)	<0.001	2.32 (1.81, 2.84)	<0.001
	20–39	1.26 (1.14, 1.37)		1.85 (1.82, 1.89)		5.71 (5.33, 6.10)	
	40–59	1.79 (1.16, 2.42)		2.36 (2.32, 2.40)		15.92 (15.38, 16.47)	
	≥60	1.10 (0.68, 1.52)		2.39 (2.32, 2.47)		31.15 (29.64, 32.72)	
Monthly income (million Won)							
	<1	1.22 (1.01, 1.43)	0.332	2.26 (2.17, 2.34)	0.357	13.58 (12.61, 14.56)	0.002
	1–2	1.53 (1.25, 1.81)		2.07 (2.03, 2.12)		13.37 (12.79, 13.97)	
	2–3	1.40 (1.14, 1.65)		2.03 (1.98, 2.07)		12.58 (12.03, 13.15)	
	≥3	1.27 (1.09, 1.45)		2.03 (1.99, 2.08)		12.08 (11.55, 12.62)	
Education							
	<High school	1.32 (0.95, 1.69)	0.358	2.22 (2.16, 2.27)	<0.001	14.09 (13.44, 14.76)	<0.001
	High school	1.53 (1.25, 1.81)		2.11 (2.06, 2.16)		13.32 (12.76, 13.90)	
	>High school	1.30 (1.16, 1.44)		1.94 (1.90, 1.98)		11.30 (10.85, 11.77)	
Smoking status							
	Never	1.27 (1.11, 1.43)	0.248	1.84 (1.81, 1.87)	<0.001	11.29 (10.87, 11.72)	<0.001
	Past-smoker	1.41 (1.13, 1.70)		2.28 (2.22, 2.34)		15.01 (14.21, 15.84)	
	Current-smoker	1.65 (1.26, 2.04)		2.59 (2.54, 2.65)		15.06 (14.36, 15.78)	
BMI (kg/m^2^)							
	<25	1.29 (1.15, 1.43)	0.214	2.05 (2.01, 2.08)	<0.001	12.70 (12.32, 13.08)	0.207
	25–30	1.57 (1.22, 1.92)		2.17 (2.12, 2.22)		13.04 (12.37, 13.72)	
	≥30	1.74 (1.03, 2.46)		2.08 (1.99, 2.19)		14.04 (12.43, 15.76)	
Occupational noise exposure							
	No	1.33 (1.20, 1.47)	0.243	2.04 (2.01, 2.07)	<0.001	12.40 (12.03, 12.77)	<0.001
	Yes	1.64 (1.16, 2.11)		2.37 (2.30, 2.45)		15.86 (14.89, 16.87)	
Loud noise exposure							
	No	1.34 (1.23, 1.46)	0.256	2.08 (2.05, 2.10)	<0.001	12.84 (12.49, 13.19)	<0.001
	Yes	1.95 (0.92, 2.98)		2.21 (2.04, 2.39)		13.34 (11.42, 15.43)	
Firearm noise exposure							
	No	1.36 (1.21, 1.52)	0.845	2.00 (1.96, 2.02)	<0.001	12.13 (11.74, 12.52)	<0.001
	Yes	1.34 (1.13, 1.54)		2.39 (2.33, 2.44)		15.28 (14.64, 15.93)	
Current diagnosis of hypertension							
	Normal	1.23 (1.11, 1.35)	0.031	2.06 (2.02, 2.10)	<0.001	13.98 (13.48, 14.49)	0.126
	Pre-hypertension	1.38 (1.03, 1.73)		2.27 (2.21, 2.33)		14.61 (13.94, 15.31)	
	Hypertension	2.25 (1.49, 3.02)		2.29 (2.24, 2.35)		14.90 (14.12, 15.70)	
Current diagnosis of diabetes							
	No	1.51 (0.61, 2.42)	0.206	2.02 (2.03, 2.28)	0.802	13.00 (9.36, 17.27)	0.868
	Yes	0.73 (0.18, 1.28)		1.92 (1.80, 2.06)		13.86 (12.33, 15.48)	

^a^Age-adjusted value except for age groups.

^b^t-test or Wald F-test

Table 2 shows logistic regression analysis results of hearing loss with earphone use time quintiles for different models. The trends of ORs for earphone use time are significant in all models. The crude percentage of hearing loss is 8.1% in the lowest use time quintile and increases to 16.2% in the highest quintile. In the fully adjusted model (model C), OR for hearing loss of the highest quintile was 2.71 (95% CI: 1.31, 5.61). A 1-hour increment in earphone use time is associated with 19% (OR = 1.19, 95% CI: 1.01, 1.41) higher odds of hearing loss in model C. The comparisons the differences between those cases with and without using earphone are available in S1 Table of supporting files.

**Table 2:** ORs (95% CIs) for hearing loss^a^ by earphone use time (n = 1,036)

Variables		No. with hearing loss / no. of participants	(%)	Model A^b^	Model B^c^	Model C^d^
Earphone using time						
Per 1 hour increasing of using time				1.18 (1.01, 1.39)	1.14 (0.97, 1.35)	1.19 (1.01, 1.41)
Using time quintiles (min)						
	Q1 (1–25)	15/185	(8.1)	Ref.	Ref.	Ref.
	Q2 (30–50)	22/242	(9.1)	0.57 (0.23, 1.41)	0.57 (0.23, 1.45)	0.59 (0.16, 2.13)
	Q3 (60–60)	22/301	(7.3)	0.81 (0.34, 1.89)	0.81 (0.35, 1.90)	1.09 (0.53, 2.21)
	Q4 (70–120)	19/179	(10.6)	1.71 (0.64, 4.57)	1.66 (0.61, 4.51)	2.25 (0.97, 5.20)
	Q5 (150–720)	21/130	(16.2)	2.04 (0.84, 4.92)	1.86 (0.75, 4.57)	2.71 (1.31, 5.61)
p for trend				0.015	0.025	0.006

^a^Hearing loss was defined as pure-tone average ≥ 25 dB.

^b^Model A was adjusted for age, sex, monthly income, education level, smoking status, BMI, and blood lead.

^c^Model B was adjusted for all variables included in model A and further adjusted for occupational noise, loud noise, and firearm noise.

^d^Model C was adjusted for all variables included in model B and further adjusted for hypertension and diabetes.

Table 3 presents logistic regression analysis results of hearing loss with blood lead level quintiles for different models. The trends of ORs for blood lead level are significant only in the completely adjusted model (model C). The crude percentage of hearing loss is 10.6% in the lowest lead level quintile and increases to 49.6% in the highest quintile. There is a significant increase in OR after adjustment for the potential confounding variables in the highest quintile (OR = 1.52, 95% CI: 1.11, 2.10). A 1 μg/dL increment in blood lead level is associated with 43% (OR = 1.43, 95% CI: 1.03, 2.00) higher odds of hearing loss in model C.

**Table 3:** ORs (95% CIs) for hearing loss^a^ by blood lead levels (n = 7,596)

Variables		No. with hearing loss / no. of participants	(%)	Model A^b^	Model B^c^	Model C^d^
Blood lead						
Per 1 μg/dL increasing of blood lead level				1.52 (1.10, 2.10)	1.50 (1.08, 2.08)	1.43 (1.03, 2.00)
Lead level quintiles (μg/dL)						
	Q1 (0.260–1.365)	160/1,511	(10.6)	Ref.	Ref.	Ref.
	Q2 (1.366–1.796)	289/1,545	(18.7)	1.12 (0.81, 1.56)	1.11 (0.79, 1.55)	1.09 (0.77, 1.54)
	Q3 (1.798–2.277)	420/1,536	(27.3)	1.35 (1.01, 1.80)	1.34 (1.00, 1.78)	1.31 (0.97, 1.77)
	Q4 (2.278–2.919)	547/1,520	(36.0)	1.48 (1.10, 1.98)	1.45 (1.08, 1.95)	1.41 (1.04, 1.92)
	Q5 (2.920–26.507)	736/1,484	(49.6)	1.61 (1.18, 2.19)	1.59 (1.16, 2.16)	1.52 (1.11, 2.10)
p for trend				0.060	0.076	0.039

^a^Hearing loss was defined as pure-tone average ≥ 25 dB.

^b^Model A was adjusted for age, sex, monthly income, education levels, smoking status, BMI, and blood lead.

^c^Model B was adjusted for all variables included in model A and further adjusted for occupational noise, loud noise, and firearm noise.

^d^Model C was adjusted for all variables included in model B and further adjusted for hypertension and diabetes.

Table 4 shows the individual and joint effects of earphone use and blood lead level on hearing loss. The OR for participants in both high groups compared with the reference group (both low earphone use time and blood lead level) is 3.23 (95% CI: 1.44, 7.27), 1.14 (95% CI: 0.42, 3.13) for participants with high lead only, and 1.79 (95% CI: 0.66, 4.85) for participants with high earphone use time only. The ORs are presented for the positive relationship between earphone use and hearing loss in strata of blood lead level and between lead exposure and hearing loss in strata of earphone use time although they were not statistically significant. The estimate of joint effect on the additive scale of earphone use time and blood lead level, the RERI, is 1.30 (95% CI: −0.83, 3.43). The observed effect is greater than the sum of the estimated effects of earphone use alone and blood lead level alone. Therefore, there is positive interaction on the additive scale, but the result is not significant. The ratio of ORs, the estimate of joint effect on the multiplicative scale, is 1.58 (95% CI: 0.42, 5.97). The observed OR is greater than the product of the estimated effects of earphone use alone and blood lead level alone. Therefore, there is a positive interaction on the multiplicative scale, but the result is not significant.

**Table 4:** ORs (95% CIs) for hearing loss^a^ by joint effect between earphone use time and blood lead level (n = 1,036)

Variables	Low lead	High lead	Lead within strata of using time
**Low using time**	Ref.	1.14 (0.42, 3.13)	1.14 (0.42, 3.13)
**High using time**	1.79 (0.66, 4.85)	3.23 (1.44, 7.27)	1.93 (0.75, 4.96)
**Using time within strata of lead**	1.79 (0.66, 4.85)	2.48 (0.97, 6.38)	

^a^Hearing loss was defined as pure-tone average ≥ 25 dB.

Measure of interaction on additive scale: RERI (95% CI) = 1.30 (−0.83 to 3.43); p = 0.232.

Measure of interaction on multiplicative scale: ratio of ORs (95% CI) = 1.58 (0.42 to 5.97); p = 0.499.

Models were adjusted for age, sex, monthly income, education levels, smoking status, BMI, occupational noise, loud noise, firearm noise, hypertension, and diabetes. Earphone use time models were further adjusted for lead, and blood lead models were further adjusted for earphone use time.

## Discussion

This study illustrates both the individual and joint effects of earphone overuse and environmental lead exposure on poor hearing thresholds by using a representative sample of the Korean population who participated in the 2010–2013 KNHANES. PTA for the highest quintile compared with the lowest quintile of earphone use time and blood lead level increases 2.71-fold and 1.52-fold, respectively, after adjusting for potential confounders. A dose-response relationship of hearing outcomes with earphone use time and blood lead level is observed. In addition, we have presented results of the joint effect of exposure to both earphone use and lead on hearing threshold, but neither additive nor multiplicative effects were statistically significant.

We controlled demographic, noise-related, and disease variables that may act as potential confounders in hearing loss research. Age is one of the major risk factors for hearing loss, and many studies have demonstrated the association between aging and hearing threshold [22–24]. Recently, mechanistic pathways that could cause age-related degeneration of hearing ability have been suggested [25]. Monthly income and education level are surrogates for social economic status (SES). Therefore, we adjusted for these variables in our statistical analysis although they are not direct factors in a poor hearing outcome. Occupational noise exposure is also a well-established risk factor. Many researchers have studied the hearing ability of laborers in the workplace [26–28]. Several countries have surveillance systems that monitor work-related noise and attest to the effects of occupational noise on hearing outcomes [29–31]. Likewise, non-occupational loud noise and firearm noise exposure also have an effect on hearing threshold. Choi et al. mentioned that their study was limited because they did not control for various potential confounding factors such as exposure to non-occupational noise [19]. Certain types of disease status, hypertension, and diabetes mellitus satisfy the condition of confounding factor as well because they can affect poor hearing ability [32–34].

Exposure to lead and noise can affect hearing loss. Animal studies have suggested possible mechanisms for lead ototoxicity [35, 36]. Although it is not yet clear how lead affects the hearing system, chronic lead exposure is known to be toxic to the central and peripheral nervous systems. Hirata and Kosaka reported that lead exposure has an effect on the conduction function in the peripheral nervous system along auditory pathways according to auditory brain stem response test results [37]. Similarly, Jones et al. provided evidence that lead exposure could alter axonal structure and function within brain stem auditory nuclei [13]. Another possible explanation for the association of lead exposure with hearing threshold degeneration is that chronic exposure to particular toxic metals is associated with the regulation of intracellular calcium homeostasis [38], and cumulative lead exposure may lead to auditory hair cell death [19].

Table 3 presents the percentage of participants with hearing loss by blood lead level quintiles. Prevalence in the highest quintile is 49.6%, but this value does not consider age effects. Blood lead level and hearing loss prevalence generally increase with age. Thus, the crude percentage of hearing loss in each quintile is a biased result. In contrast, the percentage of participants with hearing loss in the highest quintile of earphone use time was only 16.2% (see Table 2). This amount is lower than the percentage in the lead quintile. However, this difference does not indicate that blood lead has more of an effect on hearing ability than earphone use. The difference shows instead that most participants classified in the highest quintile of earphone use time are not older but younger participants.

This study has the advantage that our data are from a representative sample of the Korean population and hence permit generalization of our findings. Evidence for the individual effects of earphone use and lead exposure on hearing ability has accumulated over recent decades, but most studies have been limited to animal studies or small participant sample sizes [7, 8, 35, 36]. Few epidemiologic studies have been conducted on the general population.

This is the first study in Korea that investigates the joint effect of exposure to both earphone overuse and low-level environmental lead on increased hearing threshold. The possibility of interactions between risk factors may exist when participants are exposed to complex risk factors. For example, poor hearing ability results from the interaction between cadmium and lead exposure [39]. Moreover, there is a positive interaction between work-related noise and both organic solvents and heavy metals used in workplace [19]. In our study, however, there is no evidence for a positive interaction. The exposure level in general population is relatively lower than that of the occupational environment, it may be not enough for inducing interaction between earphone use and environmental lead exposure. All of participants in our study did not exceed the safety standard lead level of Occupational Safety and Health Administration (38.6 μg/dL).

Previous studies that observed hearing loss from exposure to both noise and heavy metals among industrial workers have several limitations compared with our study. First, occupational noise exposure is the major risk factor leading to hearing loss in the industrial workplace. Previous studies define noise level generated in the workplace as over 85 dB [19, 40, 41] that dominates the effect on work-related hearing loss. Therefore, it is difficult to demonstrate the presence of other risk factors that may lead to hearing loss except for occupational noise exposure. However, the general population receives less of an impact from noise than individuals exposed to noise in the workplace; therefore, it is relatively easier to identify an association with risk factors other than noise, such as lead exposure. Second, the exposure level to a risk factor in the workplace is high. Previous studies showed that the prevalence of hearing loss in laborers in workplace is about 70% [19, 42], but our results are much lower for the general Korean population. This difference indicates that work environments provide higher exposure levels to noise and heavy metals than other environments in the general population. Therefore, it is difficult to generalize laborer results to the general population although they indicate that noise and heavy metal exposure have a significant effect on hearing loss. Our participants were exposed to various and relatively low levels of noise and lead; therefore, this indicates that not only high amounts of noise and lead exposure but also various levels of noise and extremely low levels of lead exposure can affect hearing loss. In other words, our results can be applied at the population level. Third, the study population in the workplace is limited to a particular age and gender. The workers were mostly men in their 40s. Results from a limited study population are difficult to generalize to a nationwide population for the same reason provided in the second item of this list. Our study considered both male and female subjects over a wide range of ages, and so our results can be generalized.

Another strength of our study is the identification of the dose-response relationship between exposure level and hearing loss outcome. Researchers in a previous study considered heavy metal exposure as only exposed or not exposed because the data did not include the amount of exposure level, which was mentioned as a limitation of their study [19]. However, our study used the exposure levels of earphone use time and blood lead provided from KNHANES, and thus we could identify a dose-response relationship.

Our results are also strengthened by the adjustment for potential confounders related to noise. We sampled the general population, not in laborers in the workplace, so our participants may have been exposed to more non-occupational noise such as recreational or firearm noise. According to previous studies, non-occupational noise exposure that causes noise-induced hearing loss is as follows: gunshot >87 dB [43]; motor sports >90 dB [43]; rock concert >120–140 dB [44]; night club >95 dB [45]; Karaoke >95 dB (Kim, 2013) etc. Therefore, we adjusted for exposure to loud noise related to recreational and firearm noise, and then we also found statistically significant results.

The limitations of this study should be considered. First, we could not account for the variable of medication-related hearing loss. The study population included cases of lower hearing threshold from medications even though they were exposed to high-level noise and/or lead. Therefore, the results in the present study may underestimate the true values. Second, we used only the frequency of earphone use and could not account for the volume of earphone sound. Sound volume could have an effect on hearing ability as well, and louder earphone use may cause more hearing loss. Kwak et al. considered the sound level by selecting a preferred volume level [46]. We recommend that follow-up studies use this method. Third, blood lead level may not be an appropriate indicator of cumulative lead exposure. Environmental lead affects humans over a long period of time. Therefore, it is more appropriate to analyze the bone or the hair lead level, which provides a more reasonable estimate than the blood lead level. For this reason, Park et al. used bone lead levels to identify the relationship between cumulative lead exposure and hearing threshold [47]. However, we used blood lead levels instead of bone lead levels because the present dataset does not provide bone lead level data. Fourth, we did not consider the protective effect of selenium on hearing ability. Hung et al. reported that participants with high selenium levels had better auditory function [48]. As selenium has a good effect on ear, people with high selenium level could have a protective effect. Therefore, follow-up studies of hearing loss should consider the effects of selenium. Finally, the present study is a cross-sectional study so we cannot be sure of causal inference between exposures and hearing loss in our results.

## Conclusions

This study provide evidence that earphone use and low-level lead exposure may increase the risk of hearing loss. The joint effect of combined exposure to earphone and lead was observed, but the results were not statistically significant in both additive and multiplicative scale. These outcomes suggest that the interaction between noise and lead exposure in general population should be further investigated in follow-up studies.

## Supporting Information

S1 Fig The estimated hearing threshold of 0.5, 1, 2, 3, and 4 kHz by lead quintiles. Models were adjusted for age, occupational noise exposure, loud noise exposure, and firearm noise exposure.
(TIF)

S1 File The raw data of the Korea National Health and Nutrition Examination Survey in 2010. This data did not contain the information about audiometric measurements.
(XLSX)

S2 File The raw data of the Korea National Health and Nutrition Examination Survey in 2010. This data contains the information about audiometric measurement.
(XLSX)

S3 File The raw data of the Korea National Health and Nutrition Examination Survey in 2011. This data did not contain the information about audiometric measurements.
(XLSX)

S4 File The raw data of the Korea National Health and Nutrition Examination Survey in 2011. This data contains the information about audiometric measurement.
(XLSX)

S5 File The raw data of the Korea National Health and Nutrition Examination Survey in 2012. This data did not contain the information about audiometric measurements.
(XLSX)

S6 File The raw data of the Korea National Health and Nutrition Examination Survey in 2012. This data contains the information about audiometric measurement.
(XLSX)

S7 File The raw data of the Korea National Health and Nutrition Examination Survey in 2013. (XLSX)

S1 Table Comparison the differences between those cases with and without using earphone. (DOCX)

## References

[1] Bainbridge KE, Hoffman HJ, Cowie CC. Diabetes and hearing impairment in the United States: audiometric evidence from the National Health and Nutrition Examination Survey, 1999 to 2004. Annals of internal medicine. 2008 7 1;149(1):1–10. Pubmed Central PMCID: 2803029. 18559825 10.7326/0003-4819-149-1-200807010-00231 PMC2803029

[2] Stevens G, Flaxman S, Brunskill E, Mascarenhas M, Mathers CD, Finucane M. Global and regional hearing impairment prevalence: an analysis of 42 studies in 29 countries. The European Journal of Public Health. 2013;23(1):146–52. 10.1093/eurpub/ckr176 22197756

[3] Agrawal Y, Platz EA, Niparko JK. Risk factors for hearing loss in US adults: data from the National Health and Nutrition Examination Survey, 1999 to 2002. Otology & neurotology: official publication of the American Otological Society, American Neurotology Society [and] European Academy of Otology and Neurotology. 2009 2;30(2):139–45. 10.1097/MAO.0b013e318192483c 19092714

[4] Daniel E. Noise and hearing loss: a review. The Journal of school health. 2007 5;77(5):225–31. 10.1111/j.1746-1561.2007.00197.x 17430434

[5] Hong HN, Kang TH, Hong BN. Survey on the use of MP3 Players of high school students and the effect to their hearing thresholds. The journal of the acoustical society of Korea. 2013;32(1):56–63.

[6] Hodgetts W, Szarko R, Rieger J. What is the influence of background noise and exercise on the listening levels of iPod users? Int J Audiol. 2009;48(12):825–32. English. 10.3109/14992020903082104 20017679

[7] Muchnik C, Amir N, Shabtai E, Kaplan-Neeman R. Preferred listening levels of personal listening devices in young teenagers: Self reports and physical measurements. Int J Audiol. 2012 4;51(4):287–93. English. 10.3109/14992027.2011.631590 22122401

[8] Vogel I, Verschuure H, van der Ploeg CPB, Brug J, Raat H. Adolescents and MP3 Players: Too Many Risks, Too Few Precautions. Pediatrics. 2009 6;123(6):E953–E8. English. 10.1542/peds.2008-3179 19482747

[9] Vyskocil A, Truchon G, Leroux T, Lemay F, Gendron M, Gagnon F, et al A weight of evidence approach for the assessment of the ototoxic potential of industrial chemicals. Toxicology and industrial health. 2012 10;28(9):796–819. 10.1177/0748233711425067 22064681

[10] Johnson A-C. Occupational exposure to chemicals and hearing impairment-the need for a noise notation. PDF) Karolinska Institutet. 2008:1–48.

[11] Pierre Campo KM, Stefan Gabriel, Angela Mollerm Eberhard Nies, Maria Dolores Sole Gomez, Esko Toppila. Combined exposure to noise and ototoxic substances. EU-OSHA. 2009.

[12] Fuente A, Slade MD, Taylor T, Morata TC, Keith RW, Sparer J, et al Peripheral and central auditory dysfunction induced by occupational exposure to organic solvents. Journal of occupational and environmental medicine / American College of Occupational and Environmental Medicine. 2009 10;51(10):1202–11. 10.1097/JOM.0b013e3181bae17c 19786896

[13] Jones LG, Prins J, Park S, Walton JP, Luebke AE, Lurie DI. Lead exposure during development results in increased neurofilament phosphorylation, neuritic beading, and temporal processing deficits within the murine auditory brainstem. The Journal of comparative neurology. 2008 2 20;506(6):1003–17. 10.1002/cne.21563 18085597

[14] Mazlan R, Saim L, Thomas A, Said R, Liyab B. Ear infection and hearing loss amongst headphone users. The Malaysian journal of medical sciences: MJMS. 2002 7;9(2):17–22. Pubmed Central PMCID: 3406203. 22844220 PMC3406203

[15] Kim MG, Hong SM, Shim HJ, Kim YD, Cha CI, Yeo SG. Hearing Threshold of Korean Adolescents Associated with the Use of Personal Music Players. Yonsei Med J. 2009 12 31;50(6):771–6. English. 10.3349/ymj.2009.50.6.771 20046416 PMC2796402

[16] Levey S, Levey T, Fligor BJ. Noise Exposure Estimates of Urban MP3 Player Users. J Speech Lang Hear R. 2011 2 1;54(1):263–77. English. 10.1044/1092-4388(2010/09-0283) 20689033

[17] Shargorodsky J, Curhan SG, Henderson E, Eavey R, Curhan GC. Heavy Metals Exposure and Hearing Loss in US Adolescents. Arch Otolaryngol. 2011 12;137(12):1177–83. English. 10.1001/archoto.2011.202 22183895

[18] Trong-neng wu C-ys, etc Effects of lead and noise exposure on hearing ability. Archives of Environmental Health. 2000;55(2). 10.1080/00039890009603396 10821511

[19] Choi YH, Kim K. Noise-induced hearing loss in Korean workers: co-exposure to organic solvents and heavy metals in nationwide industries. PloS one. 2014;9(5):e97538 Pubmed Central PMCID: 4037174. 10.1371/journal.pone.0097538 24870407 PMC4037174

[20] Knol MJ, VanderWeele TJ. Recommendations for presenting analyses of effect modification and interaction. International journal of epidemiology. 2012 4;41(2):514–20. Pubmed Central PMCID: 3324457. 10.1093/ije/dyr218 22253321 PMC3324457

[21] Hosmer DW, Lemeshow S. Confidence interval estimation of interaction. Epidemiology. 1992 9;3(5):452–6. 1391139 10.1097/00001648-199209000-00012

[22] Cruickshanks KJ, Wiley TL, Tweed TS, Klein BE, Klein R, Mares-Perlman JA, et al Prevalence of hearing loss in older adults in Beaver Dam, Wisconsin. The Epidemiology of Hearing Loss Study. American journal of epidemiology. 1998 11 1;148(9):879–86. 9801018 10.1093/oxfordjournals.aje.a009713

[23] Cruickshanks KJ, Tweed TS, Wiley TL, Klein BE, Klein R, Chappell R, et al The 5-year incidence and progression of hearing loss: the epidemiology of hearing loss study. Archives of otolaryngology—head & neck surgery. 2003 10;129(10):1041–6. 14568784 10.1001/archotol.129.10.1041

[24] Park YH, Shin SH, Byun SW, Kim JY. Age- and Gender-Related Mean Hearing Threshold in a Highly-Screened Population: The Korean National Health and Nutrition Examination Survey 2010–2012. PloS one. 2016;11(3):e0150783 Pubmed Central PMCID: 4780829. 10.1371/journal.pone.0150783 26950935 PMC4780829

[25] Yamasoba T, Lin FR, Someya S, Kashio A, Sakamoto T, Kondo K. Current concepts in age-related hearing loss: epidemiology and mechanistic pathways. Hearing research. 2013 9;303:30–8. Pubmed Central PMCID: 3723756. 10.1016/j.heares.2013.01.021 23422312 PMC3723756

[26] Stanbury M, Rafferty AP, Rosenman K. Prevalence of hearing loss and work-related noise-induced hearing loss in Michigan. Journal of occupational and environmental medicine / American College of Occupational and Environmental Medicine. 2008 1;50(1):72–9. 10.1097/JOM.0b013e31815b568c 18188084

[27] Nelson DI, Nelson RY, Concha-Barrientos M, Fingerhut M. The global burden of occupational noise-induced hearing loss. American journal of industrial medicine. 2005 12;48(6):446–58. 10.1002/ajim.20223 16299704

[28] Chang TY, Liu CS, Huang KH, Chen RY, Lai JS, Bao BY. High-frequency hearing loss, occupational noise exposure and hypertension: a cross-sectional study in male workers. Environmental health: a global access science source. 2011;10:35. Pubmed Central PMCID: 3090324. 21518430 10.1186/1476-069X-10-35 PMC3090324

[29] Wu TN, Liou SH, Shen CY, Hsu CC, Chao SL, Wang JH, et al Surveillance of noise-induced hearing loss in Taiwan, ROC: a report of the PRESS-NHL results. Preventive medicine. 1998 Jan-Feb;27(1):65–9. 9465355 10.1006/pmed.1997.0238

[30] Reilly MJ, Rosenman KD, Kalinowski DJ. Occupational noise-induced hearing loss surveillance in Michigan. Journal of occupational and environmental medicine / American College of Occupational and Environmental Medicine. 1998 8;40(8):667–74. 10.1097/00043764-199808000-00002 9729748

[31] Money A, Carder M, Turner S, Hussey L, Agius R. Surveillance for work-related audiological disease in the UK: 1998–2006. Occupational medicine. 2011 6;61(4):226–33. 10.1093/occmed/kqr047 21622911

[32] Horikawa C, Kodama S, Tanaka S, Fujihara K, Hirasawa R, Yachi Y, et al Diabetes and risk of hearing impairment in adults: a meta-analysis. The Journal of clinical endocrinology and metabolism. 2013 1;98(1):51–8. 10.1210/jc.2012-2119 23150692

[33] Duck SW, Prazma J, Bennett PS, Pillsbury HC. Interaction between hypertension and diabetes mellitus in the pathogenesis of sensorineural hearing loss. Laryngoscope. 1997 12;107(12):1596–605. English. 9396671 10.1097/00005537-199712000-00004

[34] Narlawar UW, Surjuse BG, Thakre SS. Hypertension and hearing impairment in workers of iron and steel industry. Indian journal of physiology and pharmacology. 2006 Jan-Mar;50(1):60–6. 16850905

[35] Yamamura K, Terayama K, Yamamoto N, Kohyama A, Kishi R. Effects of acute lead acetate exposure on adult guinea pigs: electrophysiological study of the inner ear. Fundamental and applied toxicology: official journal of the Society of Toxicology. 1989 10;13(3):509–15. 2612783 10.1016/0272-0590(89)90287-x

[36] Lasky RE, Maier MM, Snodgrass EB, Hecox KE, Laughlin NK. The effects of lead on otoacoustic emissions and auditory evoked potentials in monkeys. Neurotoxicology and teratology. 1995 Nov-Dec;17(6):633–44. 8747745 10.1016/0892-0362(95)02006-3

[37] Hirata M, Kosaka H. Effects of lead exposure on neurophysiological parameters. Environmental research. 1993 10;63(1):60–9. 10.1006/enrs.1993.1127 8404776

[38] Sabolic I. Common mechanisms in nephropathy induced by toxic metals. Nephron Physiology. 2006;104(3):p107–14. 10.1159/000095539 16940748

[39] Choi YH, Hu H, Mukherjee B, Miller J, Park SK. Environmental cadmium and lead exposures and hearing loss in U.S. adults: the National Health and Nutrition Examination Survey, 1999 to 2004. Environmental health perspectives. 2012 11;120(11):1544–50. Pubmed Central PMCID: 3556613. 10.1289/ehp.1104863 22851306 PMC3556613

[40] Pouryaghoub G, Mehrdad R, Mohammadi S. Interaction of smoking and occupational noise exposure on hearing loss: a cross-sectional study. BMC public health. 2007;7:137 Pubmed Central PMCID: 1925081. 10.1186/1471-2458-7-137 17605828 PMC1925081

[41] Yang HY, Shie RH, Chen PC. Hearing loss in workers exposed to epoxy adhesives and noise: a cross-sectional study. BMJ open. 2016;6(2):e010533 Pubmed Central PMCID: 4762098. 10.1136/bmjopen-2015-010533 26892792 PMC4762098

[42] Girard SA, Leroux T, Courteau M, Picard M, Turcotte F, Richer O. Occupational noise exposure and noise-induced hearing loss are associated with work-related injuries leading to admission to hospital. Injury prevention: journal of the International Society for Child and Adolescent Injury Prevention. 2015 4;21(e1):e88–92. 24639292 10.1136/injuryprev-2013-040828

[43] Jokitulppo J. Estimated leisure-time noise exposure and hearing symptoms in a finnish urban adult population. Noise & health. 2003 Jan-Mar;5(17):53–62. 12537835

[44] Sadhra S, Jackson CA, Ryder T, Brown MJ. Noise exposure and hearing loss among student employees working in university entertainment venues. Ann Occup Hyg. 2002 7;46(5):455–63. English. 12176760

[45] Gallagher G. Hot music, high noise, and hurt ears: Are teens and young adults trading hearing ability for high volume. Hearing Journal. 1989;42:7–11.

[46] Kwak HW, Kim NH. Study on relations among use of earphones, stress, and hearing threshold in university students. Journal of Korean Public Health Nursing. 2012;26(1):126–36.

[47] Park SK, Elmarsafawy S, Mukherjee B, Spiro A 3rd, Vokonas PS, Nie H, et al Cumulative lead exposure and age-related hearing loss: the VA Normative Aging Study. Hearing research. 2010 10 1;269(1–2):48–55. Pubmed Central PMCID: 2934752. 10.1016/j.heares.2010.07.004 20638461 PMC2934752

[48] Chuang HY, Kuo CH, Chiu YW, Ho CK, Chen CJ, Wu TN. A case-control study on the relationship of hearing function and blood concentrations of lead, manganese, arsenic, and selenium. The Science of the total environment. 2007 11 15;387(1–3):79–85. 10.1016/j.scitotenv.2007.07.032 17764724

