# Supplementary figures and images for: The Effects of Earphone Use and Environmental Lead Exposure on Hearing Loss in the Korean Population: Data Analysis of the Korea National Health and Nutrition Examination Survey (KNHANES), 2010–2013
Source: PLoS One. 2016 Dec 28;11(12):e0168718. doi: 10.1371/journal.pone.0168718 (PMC5193416; doi:10.1371/journal.pone.0168718)

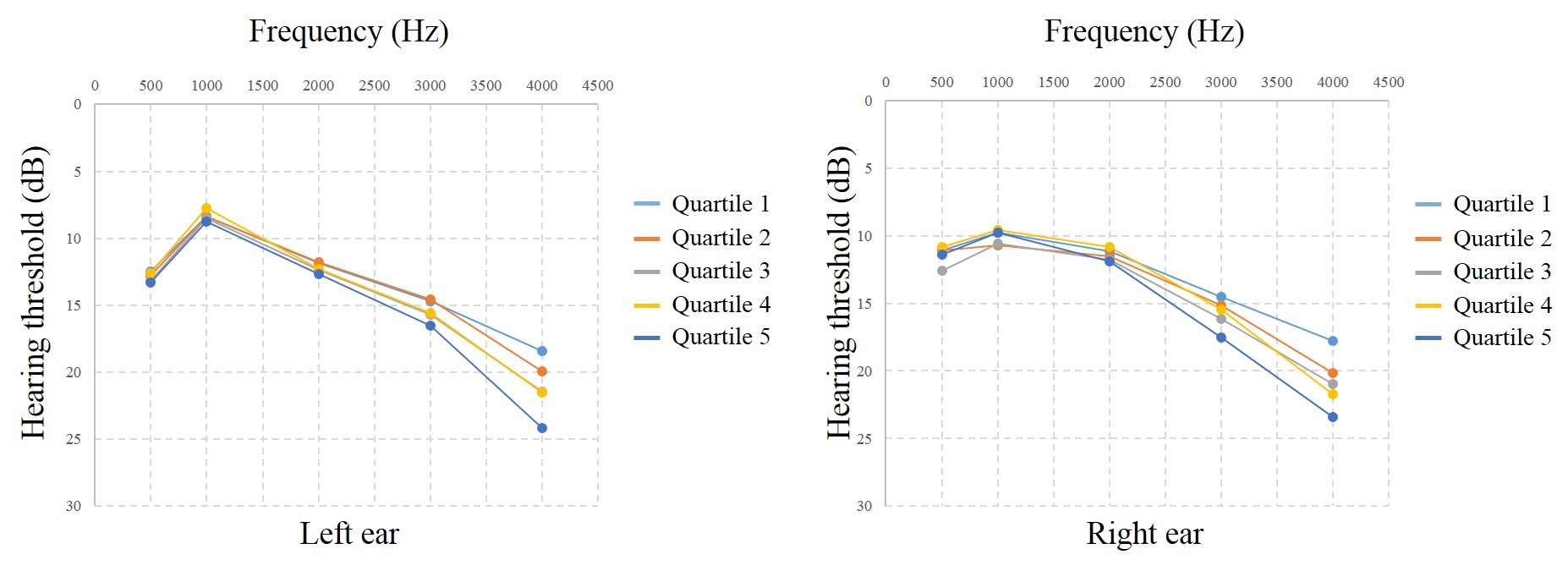

Supplement: S1 Fig — Models were adjusted for age, occupational noise exposure, loud noise exposure, and firearm noise exposure. (TIF) [file pone.0168718.s001.tif]
