# Supplementary material for: The Effects of Earphone Use and Environmental Lead Exposure on Hearing Loss in the Korean Population: Data Analysis of the Korea National Health and Nutrition Examination Survey (KNHANES), 2010–2013
Source: PLoS One. 2016 Dec 28;11(12):e0168718. doi: 10.1371/journal.pone.0168718 (PMC5193416; doi:10.1371/journal.pone.0168718)
Supplement: S1 Table — (DOCX) [file pone.0168718.s009.docx]

**S1 Table. Comparison the differences between those cases with and without using earphone**

| Variables | Total (n=7,596) | cases with using earphone (n=1,036) | cases without using earphone (n=6,560) | p-value |
| --- | --- | --- | --- | --- |
| Age, y |  |  |  | <0.001 |
| Mean±SD | 42.8±17.0 | 27.1±12.1 | 45.3±16.4 |  |
| Range | 10-87 | 12-75 | 10-87 |  |
| Male, % | 49.8 | 48.7 | 49.9 | 0.469 |
| PTA, dB |  |  |  | <0.001 |
| GM±GSD | 12.84±17.31 | 6.94±10.17 | 18.05±17.71 |  |
| Range | -10.00-110.00 | -8.33-71.67 | -10.00-110.00 |  |
| Monthly income (million Won), % |  |  |  | <0.001 |
| <1 | 14.6 | 10.0 | 15.3 |  |
| 1-2 | 26.6 | 24.0 | 27.1 |  |
| 2-3 | 28.8 | 31.9 | 28.3 |  |
| ≥3 | 30.0 | 34.1 | 29.3 |  |
| Education, % |  |  |  | <0.001 |
| <High school | 30.2 | 14.6 | 32.8 |  |
| High school | 31.3 | 27.9 | 31.8 |  |
| >High school | 38.5 | 57.5 | 35.4 |  |
| Smoking status, % |  |  |  | <0.001 |
| Never | 59.7 | 67.5 | 58.5 |  |
| Past-smoker | 18.4 | 12.5 | 19.3 |  |
| Current-smoker | 21.9 | 20.0 | 22.2 |  |
| BMI (kg/m2), % |  |  |  | <0.001 |
| <25 | 70.0 | 76.7 | 68.9 |  |
| 25-30 | 26.4 | 18.7 | 27.6 |  |
| ≥30 | 3.6 | 4.6 | 3.5 |  |
| Occupational noise exposure, % |  |  |  | 0.007 |
| No | 87.3 | 89.9 | 86.9 |  |
| Yes | 12.7 | 10.1 | 13.1 |  |
| Loud noise exposure, % |  |  |  | <0.001 |
| No | 97.8 | 95.5 | 98.1 |  |
| Yes | 2.2 | 4.5 | 1.9 |  |
| Firearm noise exposure, % |  |  |  | 0.894 |
| No | 77.0 | 76.8 | 77.0 |  |
| Yes | 23.0 | 23.2 | 23.0 |  |
| Current diagnosis of hypertension, % |  |  |  | <0.001 |
| Normal | 46 | 64.3 | 43.6 |  |
| Pre-hypertension | 25.2 | 23.3 | 25.5 |  |
| Hypertension | 28.8 | 12.4 | 30.9 |  |
| Current diagnosis of diabetes, % |  |  |  | 0.389 |
| No | 91.3 | 84.6 | 91.5 |  |
| Yes | 8.7 | 15.4 | 8.5 |  |
